# Supplementary material for: Tyramide-conjugated DNA barcodes enable signal amplification for multiparametric CODEX imaging
Source: Commun Biol. 2022 Jun 27;5:627. doi: 10.1038/s42003-022-03558-8 (PMC9234042; doi:10.1038/s42003-022-03558-8)
Supplement: Supplementary file 2 — Supplementary Information [file 42003_2022_3558_MOESM2_ESM.pdf]

Supplemental information for “Tyramide-conjugated DNA barcodes enable signal amplification for multiparametric CODEX imaging”

Paul D. Simonson*, Itzel Valencia, and Sanjay S. Patel

Department of Pathology and Laboratory Medicine, Weill Cornell Medicine, New York, NY

*Corresponding author:

Paul D. Simonson, M.D., Ph.D.

Department of Clinical Pathology and Laboratory Medicine

Weill Cornell Medicine

525 E 68th Street, Starr 702B

New York, NY 10021

pds9003@med.cornell.edu

Supplementary Figures

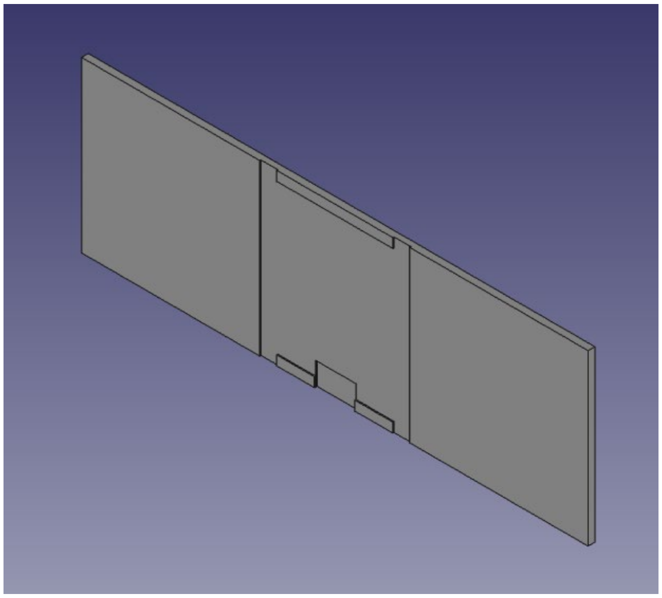

Supplementary Figure 1. Coverslip holder design for staining coverslips on a Leica Bond RX tissue autostainer. The slots at the four corners of the coverslip placement site are in place to facilitate manufacturing and ensure a good fit for the coverslip. The pocket on the side (bottom edge) facilitates lifting the coverslip out of the holder.

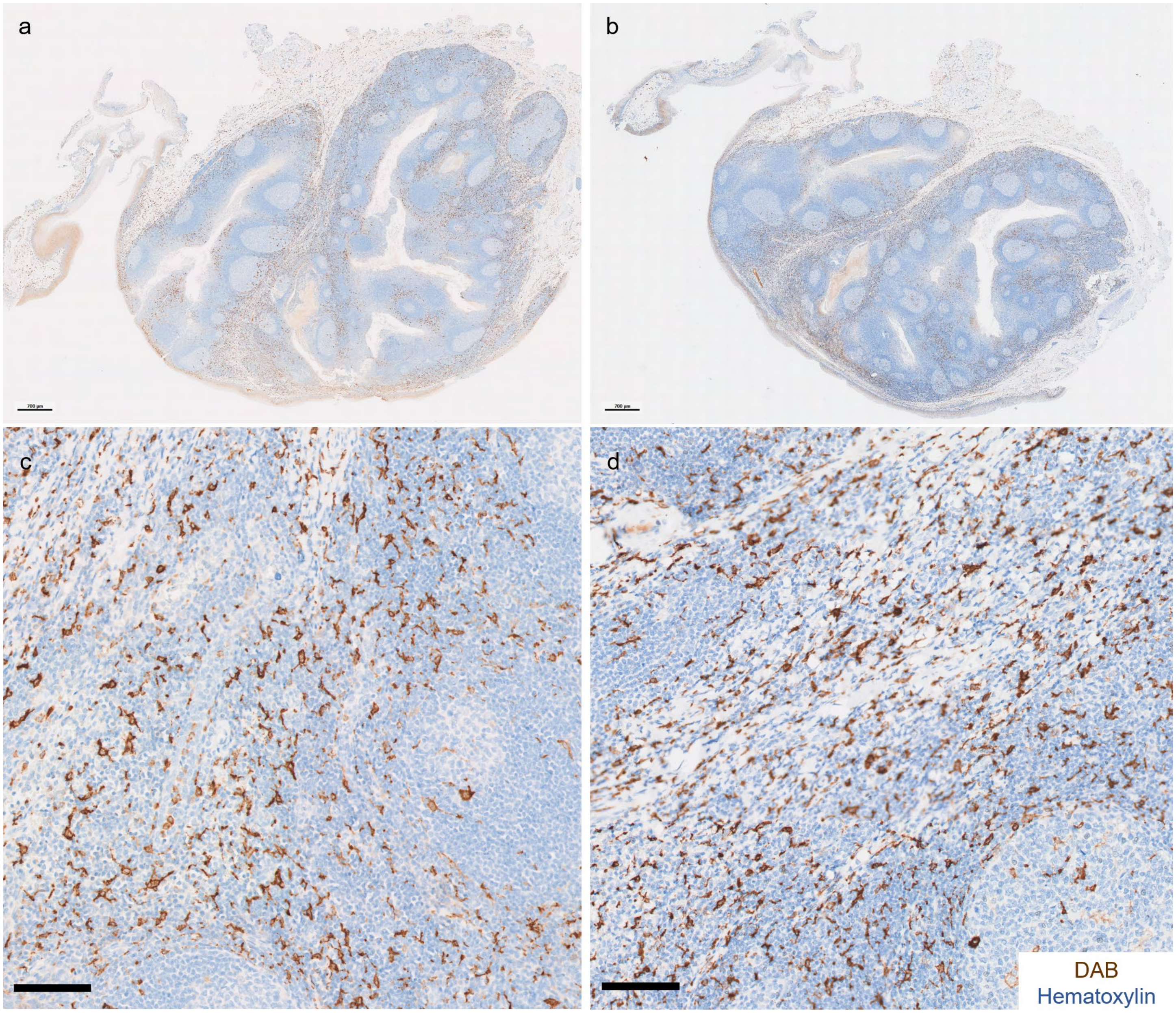

Supplementary Figure 2. Validation of staining using the custom coverslip holder. Comparison of CD163 immunohistochemical staining obtained using a glass slide (**a**, **c**) and a coverslip stained using the custom coverslip holder (**b**, **d**) and otherwise identical staining protocols. No significant qualitative difference in staining quality was identified. Slides were scanned using a Perkin-Elmer Vectra Polaris instrument at 20x magnification. Scale bars in **a** and **b** are 700 microns. Scale bars in **c** and **d** are 100 microns. DAB = 3,3'-diaminobenzidine.

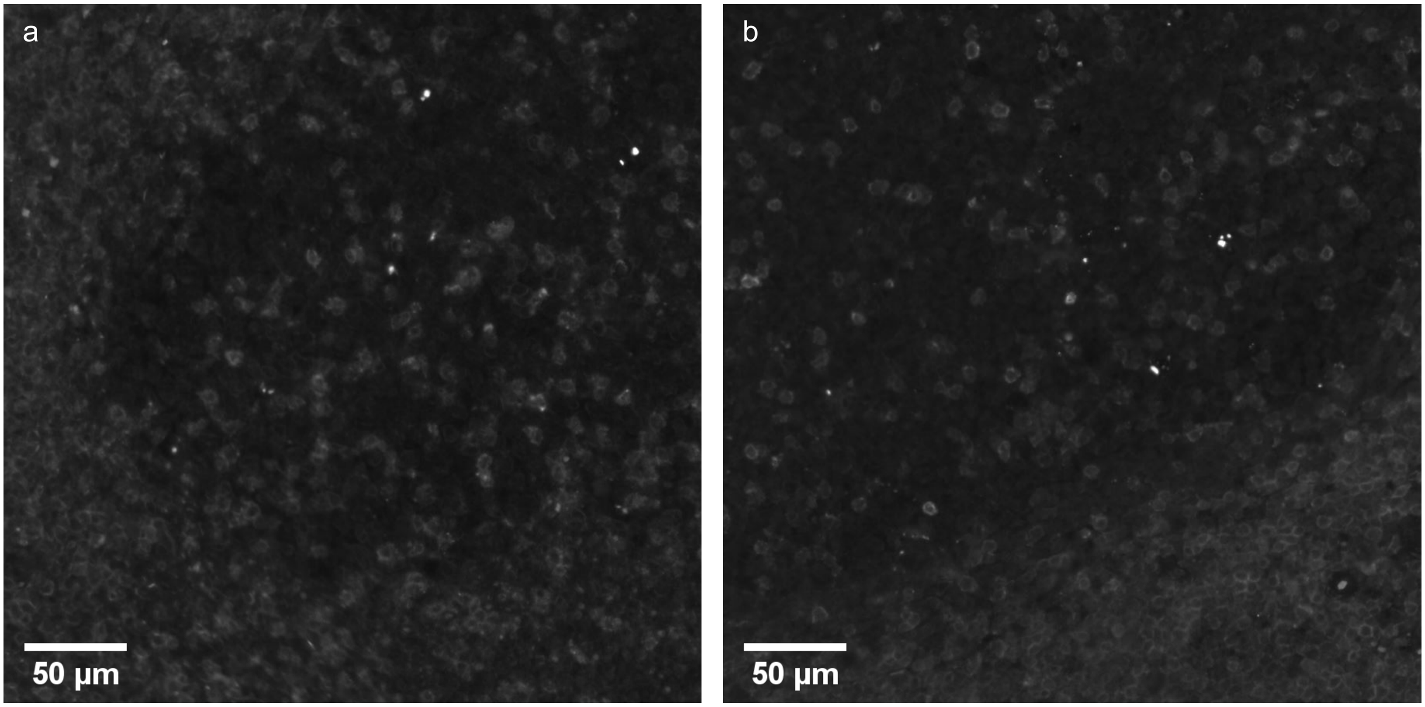

Supplementary Figure 3. Comparison of tonsil tissue on a microscope slide versus a coverslip stained with CD3e primary antibody (clone SP7, Sigma, cat. no. SAB5500058-100UL) and tyramide-barcode staining. Tonsil tissue was placed on a charged microscope slide (**a**) and a charged coverslip (**b**), which then were deparaffinized, washed (100% ethanol x3, Bond Wash solution x3), underwent antigen retrieval (Leica ER2 solution, EDTA pH 9.0) for 20 minutes at 95˚, washed 5x with Leica Bond Wash solution, incubated with Leica 3-4% hydrogen peroxide peroxidase blocking solution (5 minutes), washed 3x with Leica Bond Wash solution (0 minutes per wash), stained with CD3e (1:100 dilution, clone SP7, Sigma, cat. no. SAB5500058-100UL) primary antibody diluted in Abcam antibody diluent (Abcam, cat. no. ab64211) for 30 minutes, washed 3x with Leica Bond Wash solution (2 minutes per wash), incubated with goat anti-rabbit secondary antibody and peroxidase conjugate ready-to-use solution (Leica, 8 minutes), washed 3x with Leica Bond Wash solution (2 minutes per wash), incubated with 500 nM tyramide-barcode77 with Cy5-conjugated reporter strand in TE buffer (15 minutes), incubated 5 minutes in DAPI solution (Akoya Nuclear Stain, diluted 3 drops per 1 mL of TBS), washed 1x with Leica Bond Wash solution (2 minutes), and imaged on a Keyence BZ-X800 fluorescence microscope with 20x Nikon PlanApo lambda objective and DAPI and Cy5 appropriate filter sets. The images show no significant difference in intensity or distribution of staining when comparing the microscope slide (**a**) and coverslip (**b**).

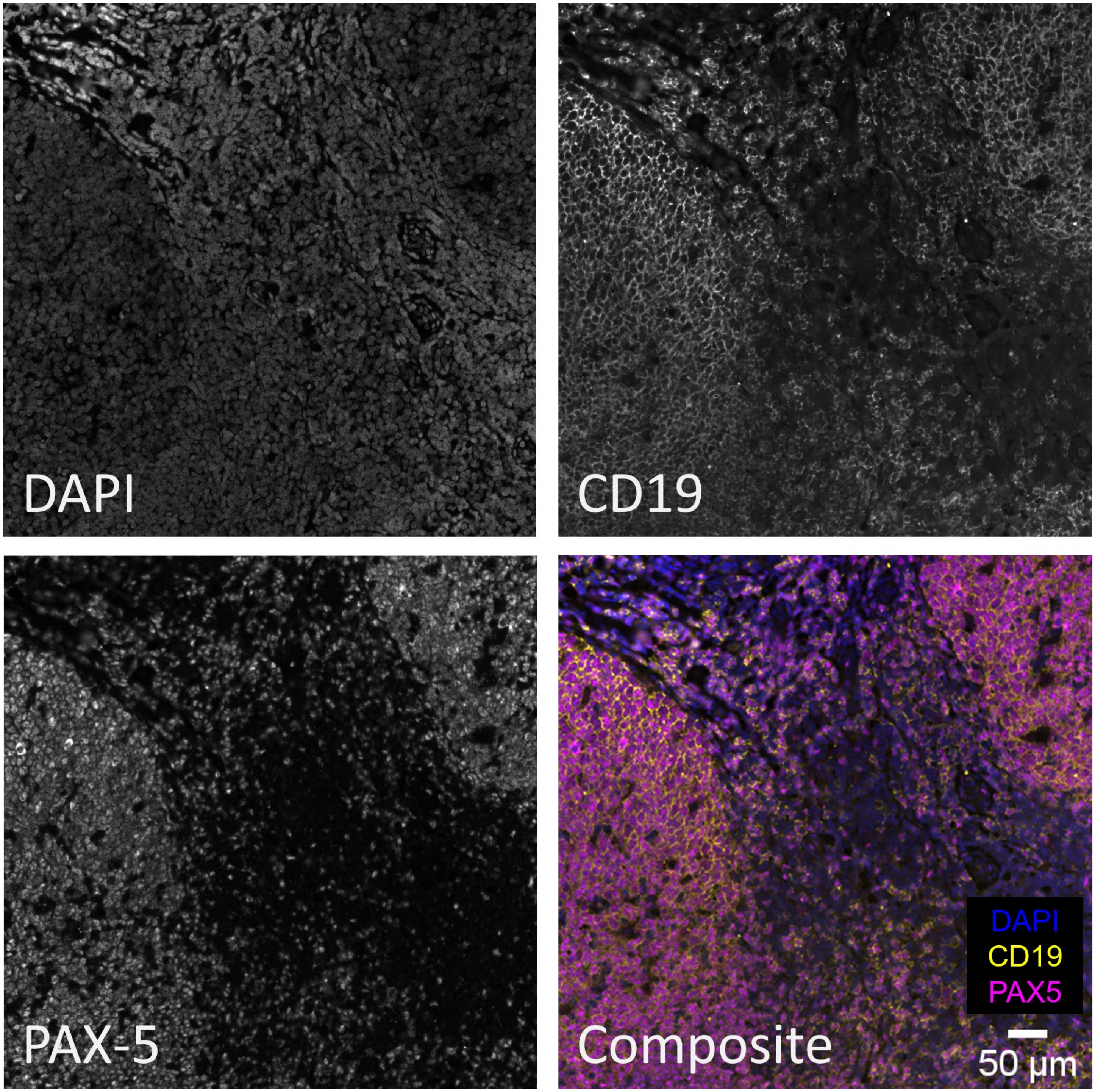

Supplementary Figure 4. Multiplexed tyramide-barcode staining of tonsil. Tonsil was stained on a Leica Bond RX autostainer with DAPI nuclear stain, mouse anti-CD19 antibody (ready-to-use solution, clone BT51E, Leica cat. no. PA0843), and mouse anti-PAX-5 antibody (1:50 dilution, clone 24/PAX-5, BD Transduction Laboratories, cat. no. 610863) primary antibodies (in different staining steps). The primary antibodies were followed by Leica rabbit anti-mouse antibodies (ready-to-use solution) and peroxidase conjugated tertiary antibodies (ready-to-use solution, Leica), which were subsequently allowed to react with tyramide-barcode-Cy3 (for CD19) and tyramide-barcode-Cy5 (for PAX-5). The composite image shows DAPI-stained nuclei in blue, CD19-stained cell membranes in yellow, and PAX-5 stained nuclei in magenta and demonstrates the appropriate co-localization of PAX-5 and CD19 in B cells. Images were captured with a

Keyence BZ-X800 microscope with 20x Nikon PlanApo 0.75 NA objective and "high resolution" camera settings. 50 µm scale bar applies to all 4 panels.

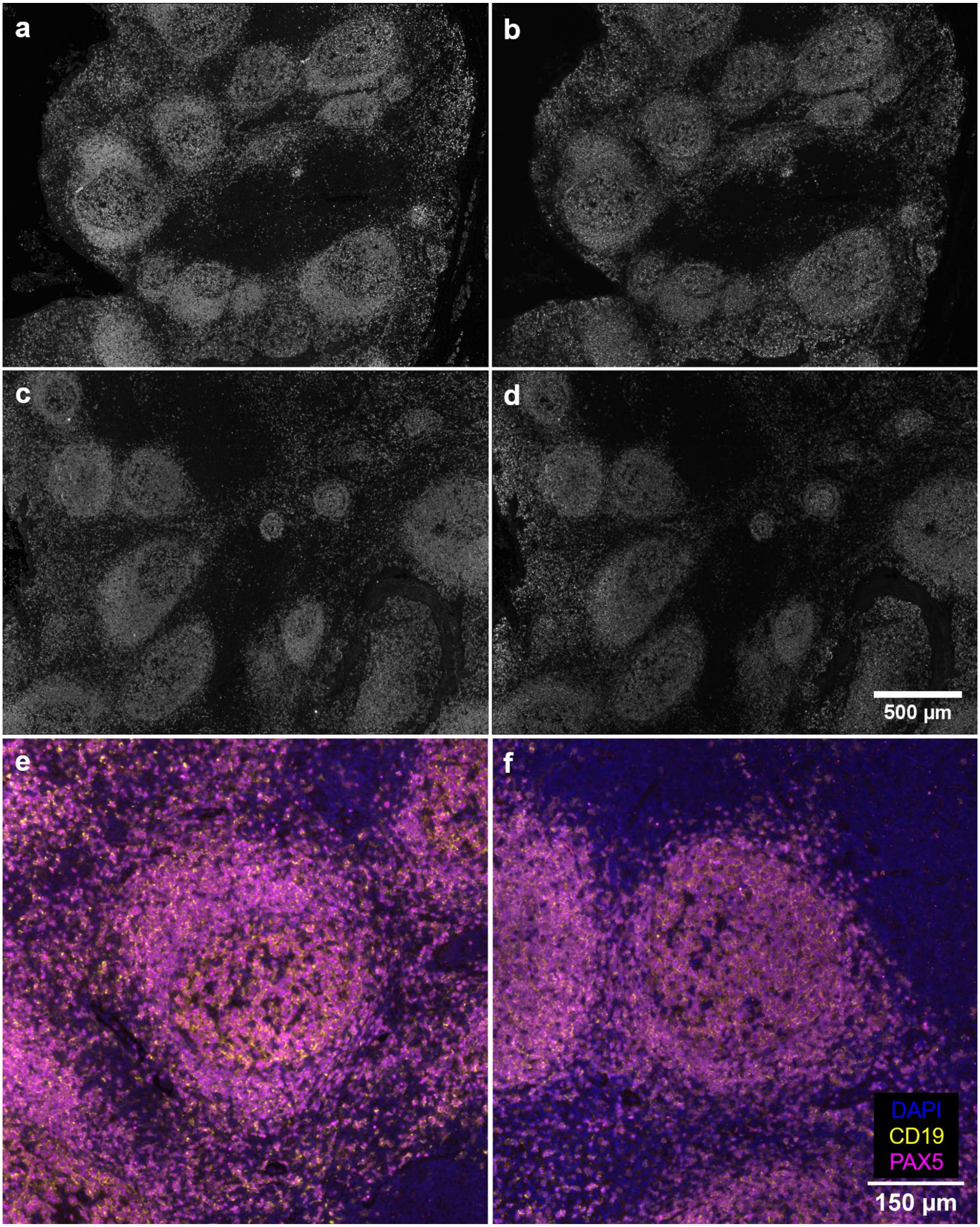

Supplementary Figure 5. Comparison of staining with tyramide-barcodes with fluorescent complementary oligomers versus staining with tyramide conjugated directly to fluorophores. Parts **a** and **b** demonstrate tonsil stained with

tyramide-barcodes, with PAX-5 highlighted with Cy3-conjugated complementary oligomer in **a**, and CD19 highlighted with Cy5-conjugated complementary oligomer in **b**. Parts **c** and **d** demonstrate tonsil stained with *tyramide conjugated directly to fluorophores*, with PAX-5 highlighted with Cy3 in **a**, and CD19 highlighted with Cy5 in **d**. In all four parts (**a**-**d**), staining is consistent with staining of B cells, found primarily in germinal centers and associated mantle and marginal zones. Part **e** demonstrates a composite image of tyramide barcode staining (parts **a** and **b**) plus DAPI staining. Part **f** demonstrates a composite image of tyramide-fluorophore staining (parts **c** and **d**), plus DAPI. The composite images show DAPI-stained nuclei in blue, CD19-stained cell membranes in yellow, and PAX-5 stained nuclei in magenta and demonstrate the appropriate co-localization of PAX-5 and CD19 in B cells. Images were captured with a Keyence BZ-X800 microscope with 20x Nikon PlanApo 0.75 NA objective and "high resolution" camera settings and equivalent acquisition times for both tyramide-barcode and tyramide-fluorophore stains. Scale bar in **d** applies to **a-d**. Scale bar in **f** applies to **e** and **f**.

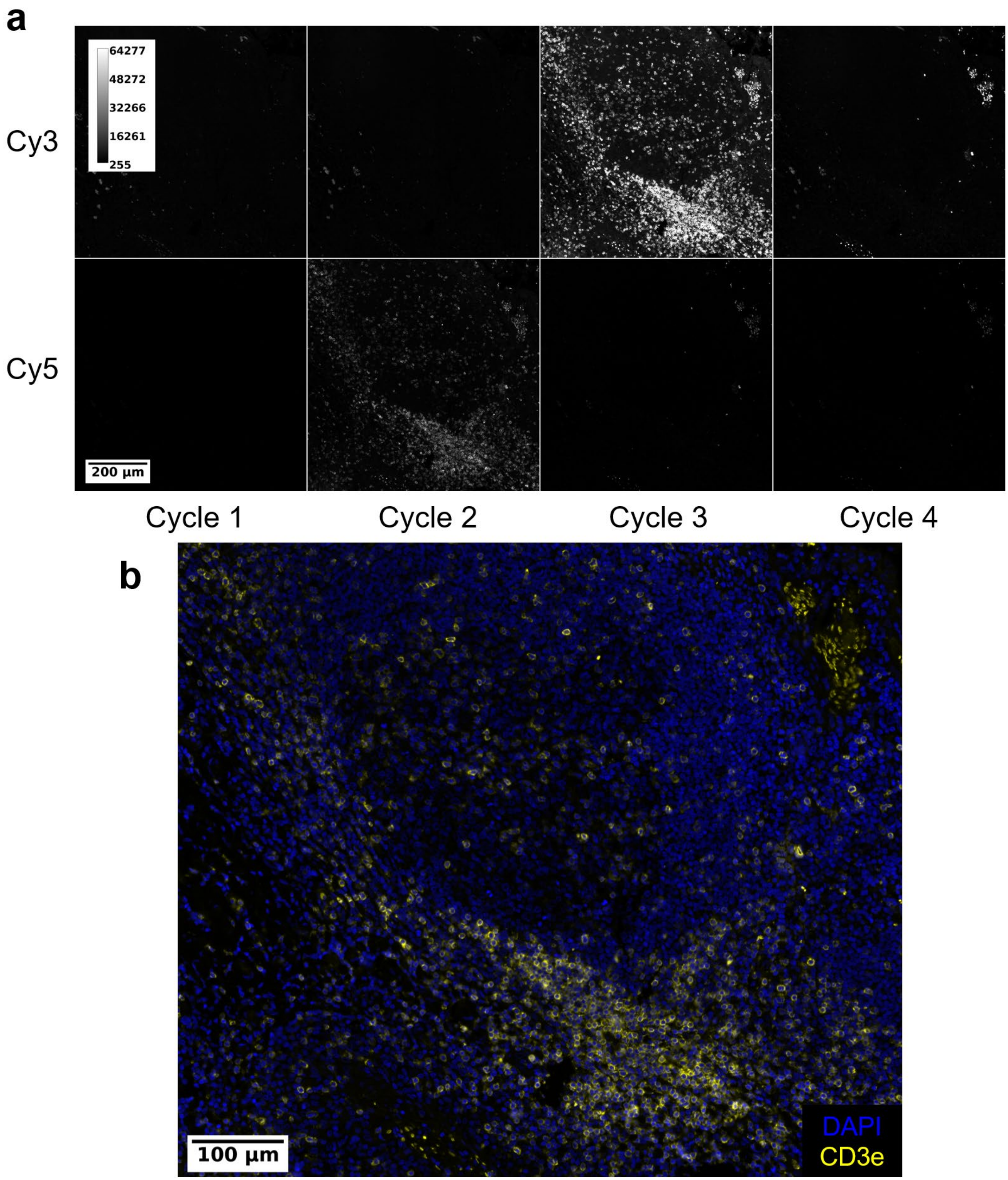

Supplementary Figure 6. Demonstration of appropriate cycling with tyramide-barcode stained tonsil using an Akoya CODEX instrument and Keyence BZ-X800 microscope. Prior to imaging, tissue was stained using a Leica Bond RX

autostainer with rabbit anti-CD3e primary antibody, peroxidase-conjugated secondary antibody, and tyramide-barcode. The tissue was then imaged on the CODEX/Keyence system using four imaging cycles; the tissue is cleared between each cycle. (**a**) In cycle 1, no complementary barcode was added; hence no staining was seen. In cycle 2, Cy5-conjugated reporter (complementary barcode) was added, with appropriate staining seen in the Cy5 imaging channel. In cycle 3, Cy3-conjugated reporter was added, with appropriate imaging seen in the Cy3 channel. In cycle 4, no reporter was added, and appropriate clearing of reporters is seen. Though appropriate clearing is seen, close inspection demonstrates very weak persistent signal in the Cy3 channel in Cycle 4 and in the Cy5 channel for Cycle 3, both demonstrating approximately 70-fold decrease from the cycles immediately prior. Cycle 4 of the Cy5 channel demonstrates a further 2-fold decrease in the residual signal, attributed to either photobleaching or further clearing of the reporter strands by the CODEX instrument. Intensity and size scales apply to all panes of part **a**. (**b**) False-colored image consists of DAPI (blue) and CD3e tyramide-barcode staining with Cy5-conjugated reporter (yellow). DAPI staining helps to demonstrate a germinal center while CD3e staining highlights T lymphocytes surrounding and scattered within the germinal center. Camera/microscope settings: 200 ms exposure time in Cy3 and Cy5 channels, 100% excitation intensity, “high resolution” imaging, Nikon 20x PlanApo lambda 0.75 NA objective. Camera pixels were saturated by the Cy3 intensity in cycle 3, demonstrating a need to further reduce the exposure time. Background subtraction and image stitching were performed using Akoya’s CODEX Processor software.

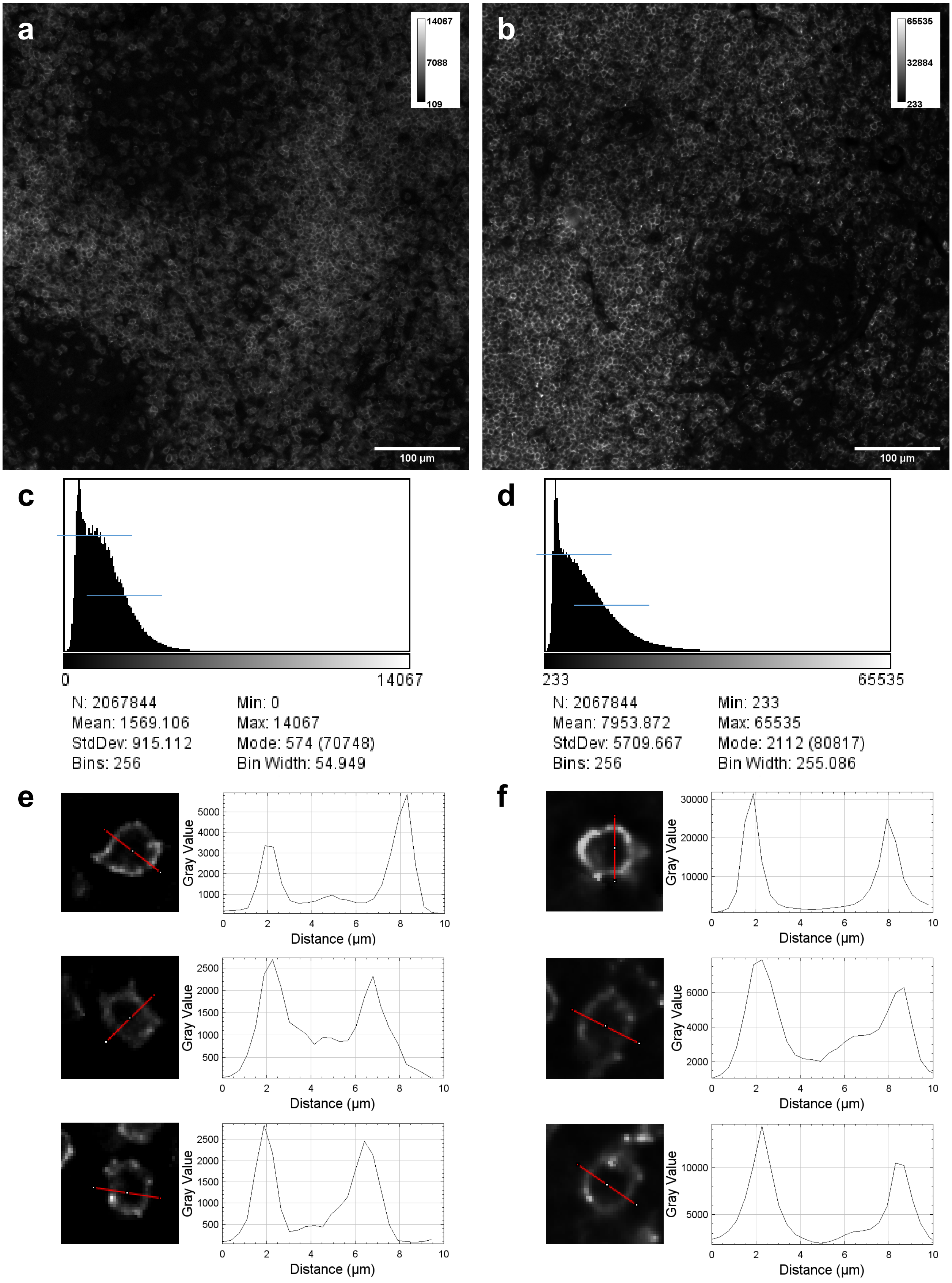
a
14067
7088
109
100 µm
b
65535
32884
233
100 µm
c
0
14067
N: 2067844
Mean: 1569.106
StdDev: 915.112
Bins: 256
Min: 0
Max: 14067
Mode: 574 (70748)
Bin Width: 54.949
d
233
65535
N: 2067844
Mean: 7953.872
StdDev: 5709.667
Bins: 256
Min: 233
Max: 65535
Mode: 2112 (80817)
Bin Width: 255.086
e
Gray Value
Distance (µm)
f
Gray Value
Distance (µm)

Supplementary Figure 7. Comparison of tissue stained by conventional CODEX staining and tyramide-barcode CODEX staining. To verify that the method actually amplifies signal, tonsil tissue was stained with barcode-conjugated antibody for CD3e (1:200 dilution, Akoya Biosciences, cat. no. 4450030). This was followed by CODEX imaging using the corresponding Cy5-conjugated complementary DNA oligomer (panel **a**). For comparison, a section of the same tissue was stained with the same primary antibody, but then followed by incubation with secondary antibody with attached peroxidase, then incubation with tyramide-conjugated barcode, followed by CODEX imaging using Cy5-conjugated complementary oligomer specific for the tyramide-barcode (panel **b**). Panels **a** and **b** demonstrate appropriate localization of staining to T cells in the images, albeit with very different fluorescence intensity distributions, which intensity distributions are also shown in parts **c** and **d** (note the scale bars of the plots). Comparison of approximate modal values and half modal values (approximately indicated by blue lines shown in **c** and **d**), demonstrates approximately 4.7-fold increase in staining intensity for the tyramide-barcode staining over regular CODEX staining. Parts **e** and **f** demonstrate cross sectional image intensity profiles for a small sample of individual cells (cropped images from parts a and b, respectively) to get a sense of imaging resolution achieved using conventional CODEX imaging (**e**) and tyramide-barcodes imaging (**f**); the imaging resolutions appear similar for the given experiments. Images were captured using an Akoya CODEX instrument and equivalent camera settings for both experiments on a Keyence BZ-X800 microscope with 20x Nikon PlanApo 0.75 NA objective. Intensity scales are in terms of camera counts.

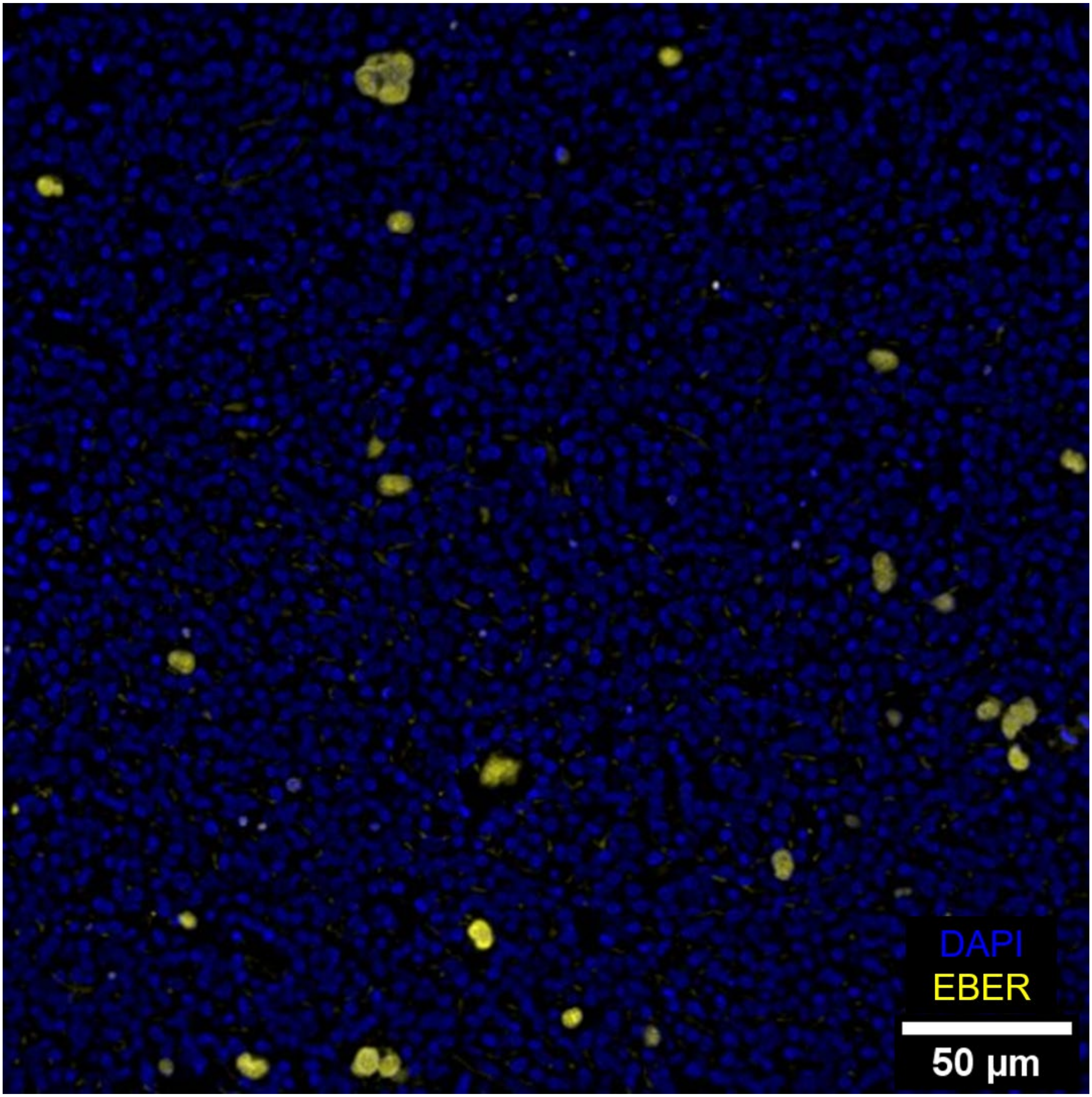

Supplementary Figure 8. EBER *in situ* hybridization (ISH) imaging using tyramide-barcode staining. Staining was performed on a Leica BondRX autostainer using EBER ISH probes conjugated to FITC, followed by sequential incubation with anti-FITC primary antibodies, secondary antibodies, peroxidase conjugated tertiary antibodies, and custom tyramide-barcodes. Imaging was performed with DAPI (shown in blue) and Cy5-conjugated complementary DNA barcodes (shown in yellow). Positive EBER staining is appropriately seen in nuclei of infiltrating large cells, consistent with EBV+ Hodgkin/Reed-Sternberg cells; some weak interstitial staining is also seen. Camera/microscope settings: 500 ms exposure in Cy5 channel, 30% excitation intensity, “high sensitivity” imaging, Nikon 20x PlanApo lambda 0.75 NA objective.

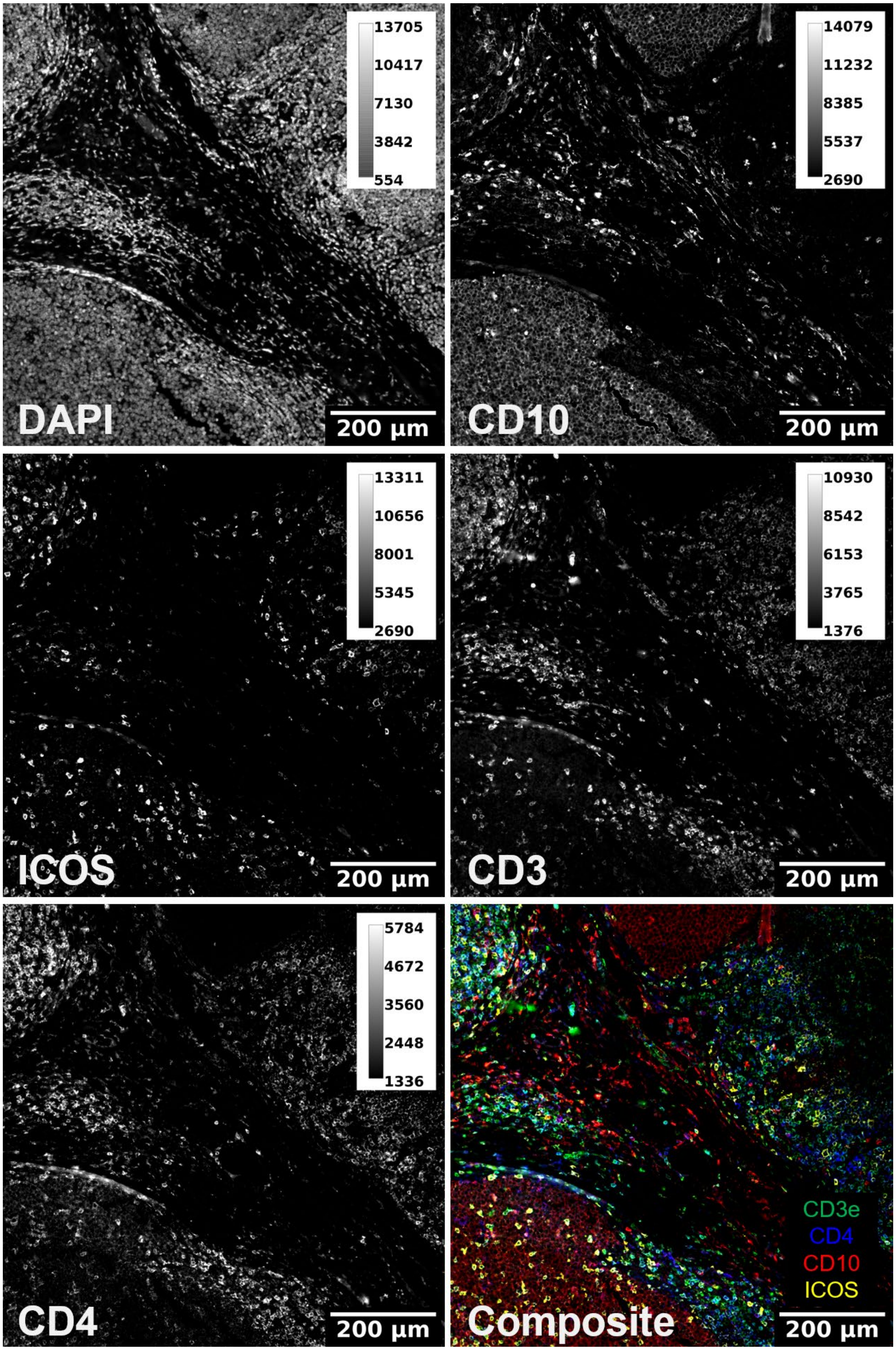

Supplementary Figure 9. Multiple antibody staining using four tyramide-barcodes in tonsil, imaged using an Akoya CODEX instrument and Keyence BZ-X800 microscope. Tissue was stained using a Leica Bond RX autostainer with

rabbit CD3e (1:100 dilution, clone SP7, Sigma), mouse CD4 (1:100 dilution, clone 4B12, Leica cat. No. NCL-L-CD4-368), mouse CD10 (1:200 dilution, clone OTI2A4, Origene cat no. CF810614) and rabbit ICOS (1:200 dilution, clone D1K2T, Cell Signaling cat. No. 89601) primary antibodies with signal amplification using tyramide-barcodes as described in above. The tissue was then imaged on the CODEX/Keyence system with Cy5-conjugated complementary DNA barcodes and 500 ms exposure time. The composite image is colored as follows: CD10 = red, ICOS = yellow, CD3e = green, and CD4 = blue. The Keyence BZ-X800 microscope used 100% excitation intensity, “high resolution” imaging, and a Nikon 20x PlanApo lambda 0.75 NA objective. Background subtraction and image stitching were performed using Akoya’s CODEX Processor software.

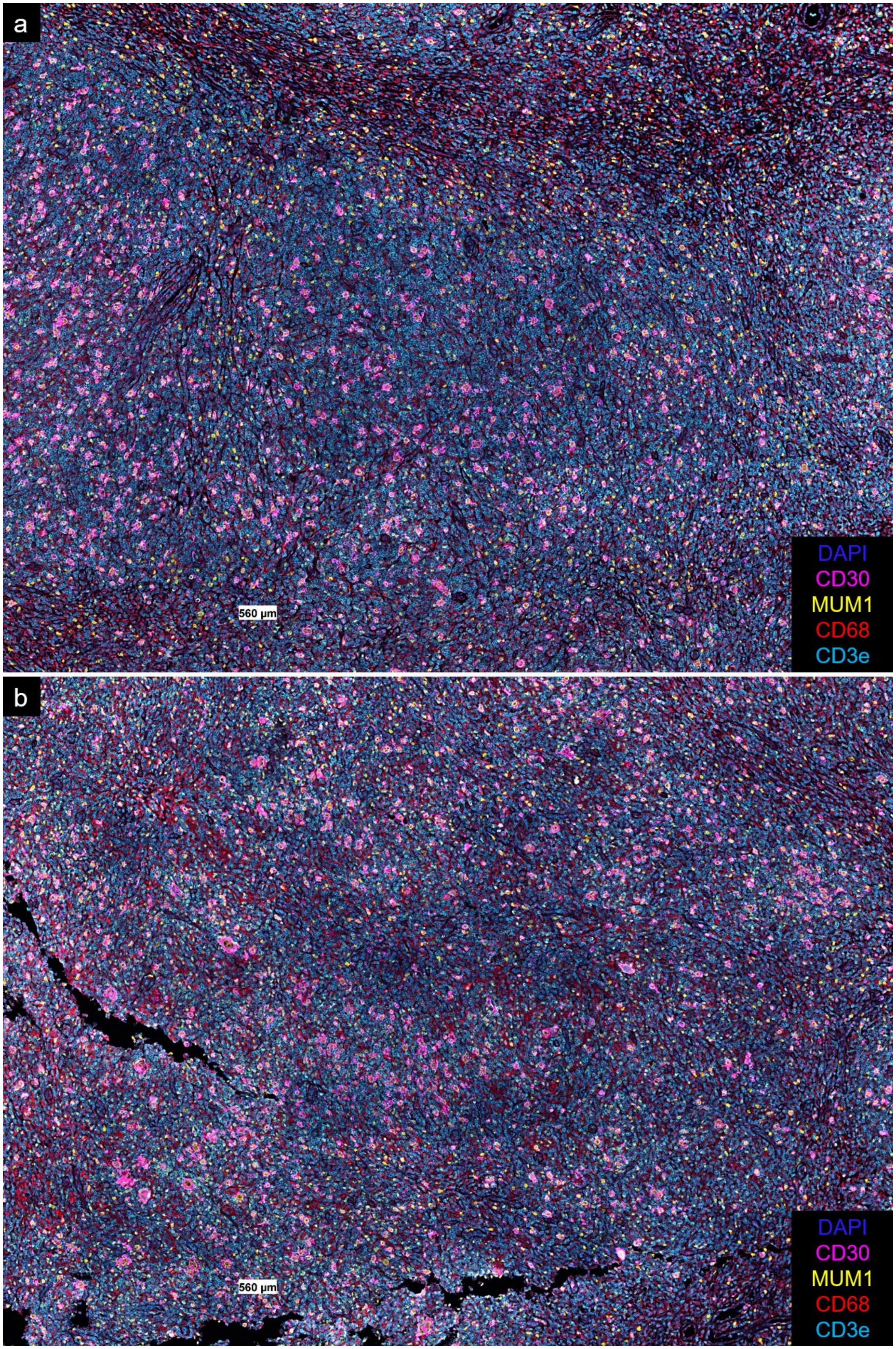

Supplementary Figure 10. Overview images of classic Hodgkin lymphoma stained with commercially available barcode-conjugated antibodies and CD30 and MUM1 stained using tyramide barcodes. Images in **a** and **b** are the two complete

regions captured by CODEX imaging. DAPI = blue, CD30 = magenta, MUM1 = yellow, CD68 = red, CD3e = cyan. See also Figure 3, which is composed of a zoomed-in region from the top image, and Supplementary Figure 11. Intensity scale bars are in terms of camera counts.

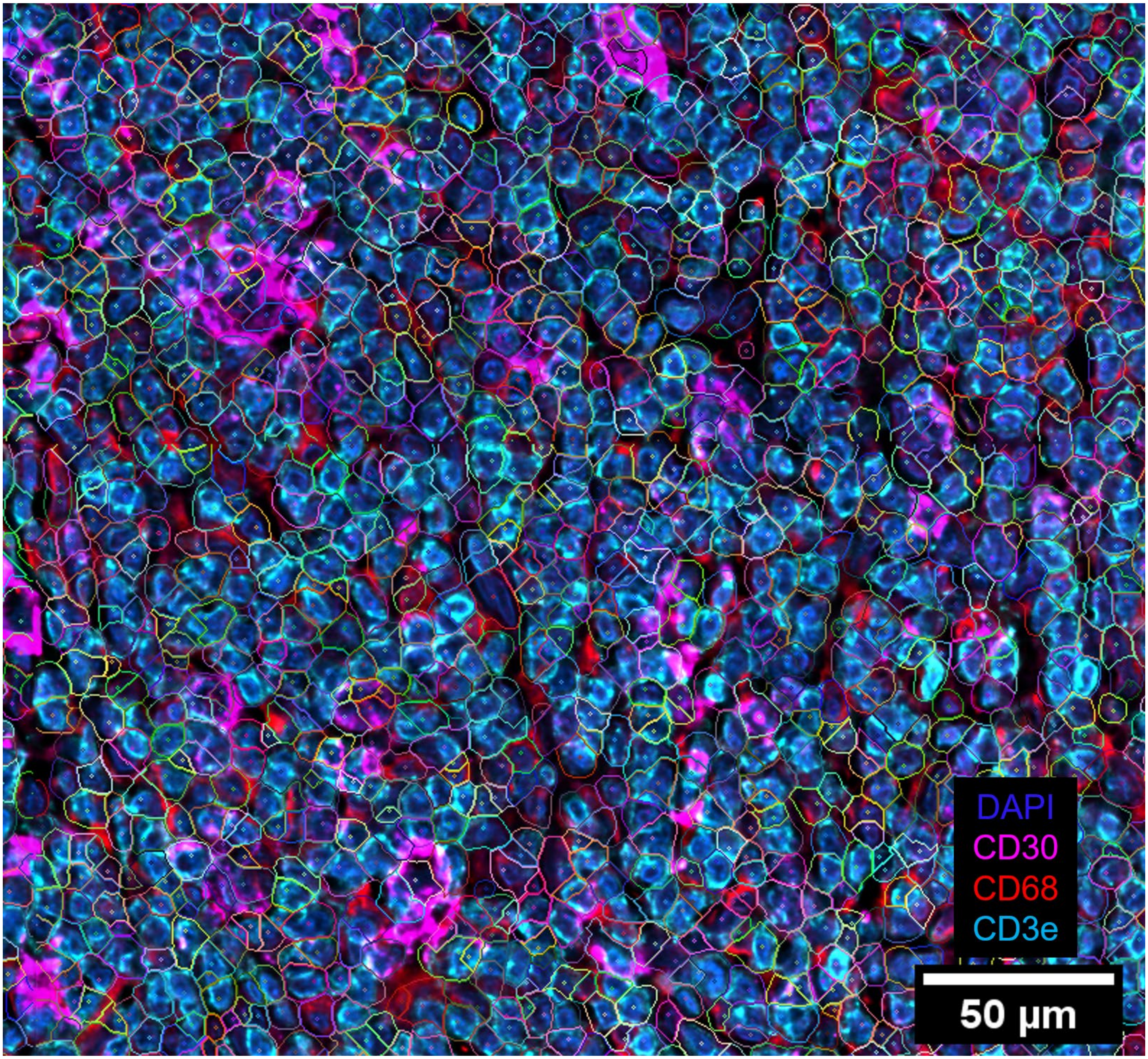

Supplementary Figure 11. Example cell segmentation in case of classic Hodgkin lymphoma. Image segmentation into cells was performed using Akoya Bioscience's CODEX Processor software and displayed here using the CODEX MAV plugin (Akoya Biosciences) for ImageJ. Cell segmentation is noted to be imperfect, particularly in the large Hodgkin cells, highlighted by CD30 staining, which are subdivided into smaller Hodgkin cell segments. DAPI = blue, CD30 = magenta, CD68 = red, CD3e = cyan.

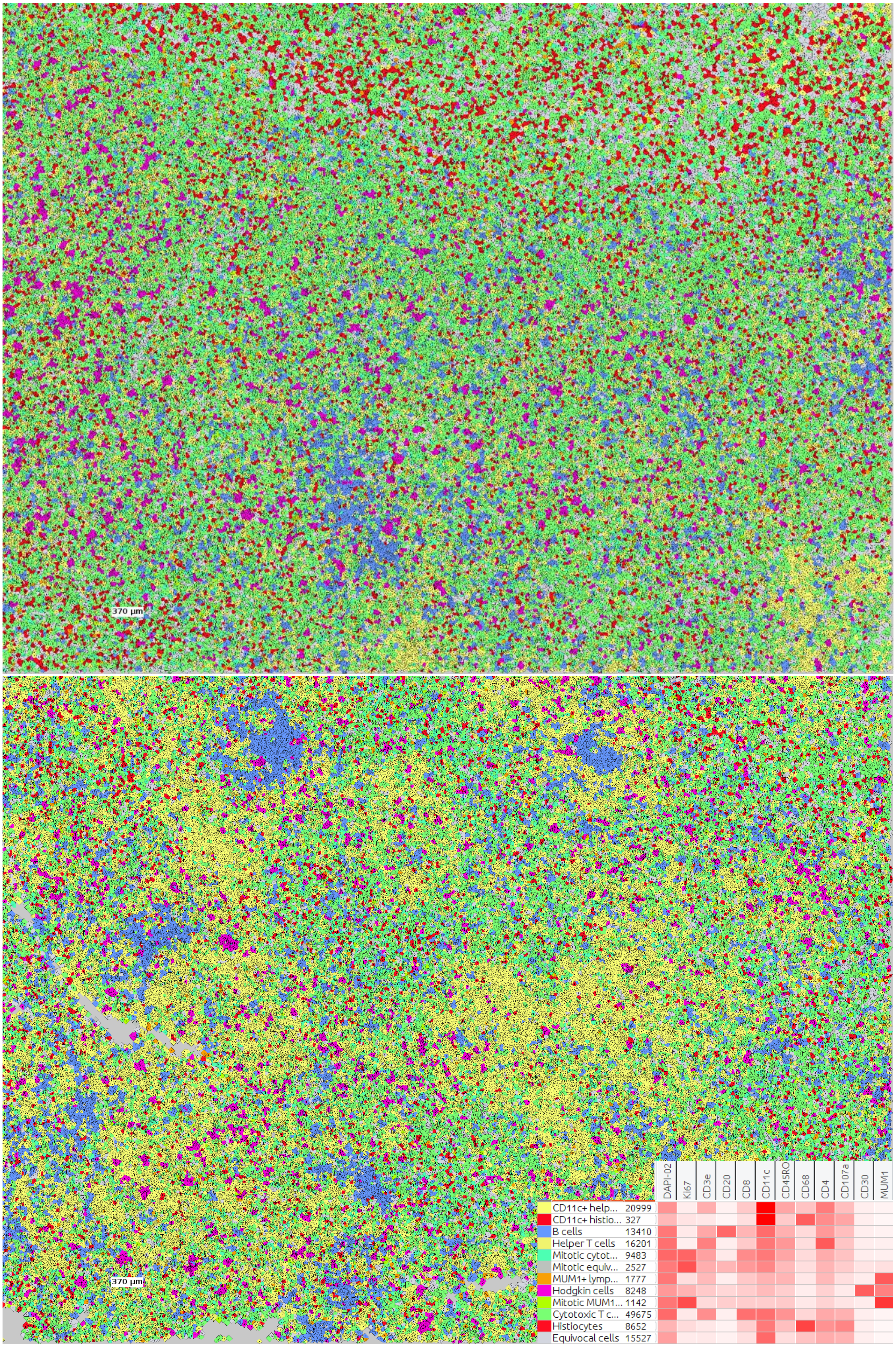
370 µm
370 µm
DAPI-02
Ki67
CD3e
CD20
CD8
CD11c
CD45RO
CD68
CD4
CD107a
CD30
MUM1
CD11c+ help... 20999
CD11c+ histio... 327
B cells 13410
Helper T cells 16201
Mitotic cytot... 9483
Mitotic equiv... 2527
MUM1+ lymp... 1777
Hodgkin cells 8248
Mitotic MUM1... 1142
Cytotoxic T c... 49675
Histiocytes 8652
Equivocal cells 15527

Supplementary Figure 12. Voronoi plots with cell classifications in both imaged regions of the example case of classic Hodgkin lymphoma (see also Supplementary Figure 10). Cell classifications were determined by using the X-shift algorithm implemented in the CODEX MAV plugin (Akoya Biosciences) for ImageJ, followed by manually combining cell clusters that are of similar immunophenotype and morphologic findings. Cells labeled as "CD11c+ helper T cells" and "CD11c+ histiocytes" are in fact most likely to be helper T cells and histiocytes that do not express CD11c but are adjacent to CD11c+ dendritic cells. Cells labeled as "equivocal" appear largely to be composed, among other cell types, of endothelial cells and stromal cells. Voronoi plots are produced using the CODEX MAV plugin. For the relative marker intensity scale for the cell types shown in the bottom right of the image, darker shades of red indicate stronger marker fluorescence intensity while lighter shades of red indicate less marker fluorescence intensity.

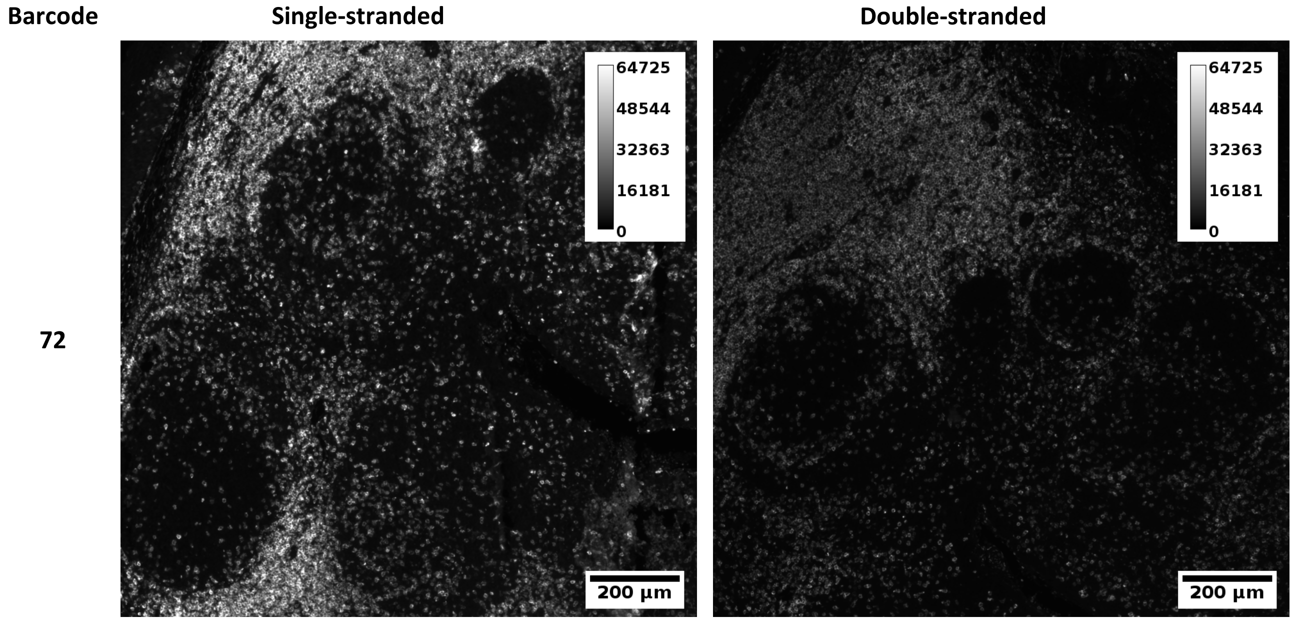

Supplementary Figure 13. Comparison of CD3e staining using single-stranded versus double-stranded tyramide-barcodes in the step involving incubation with peroxidase demonstrates that single-stranded tyramide barcodes and double-stranded tyramide barcodes perform similarly well. In this figure, results are shown for barcode "72". Comparisons for barcodes "74", "75", "76", "77", "79", and "80" are shown in Supplementary Figures 14 and 15. All images were recorded using the same camera settings, including 0.5 s camera acquisition times. See also the experiment details in Methods. Note that our early experiments failed to demonstrate staining with single-stranded tyramide barcodes for unknown reasons; however, the findings could not be replicated as later experiments all successfully demonstrated staining with single-stranded DNA barcodes. Intensity scale bars are in terms of camera counts.

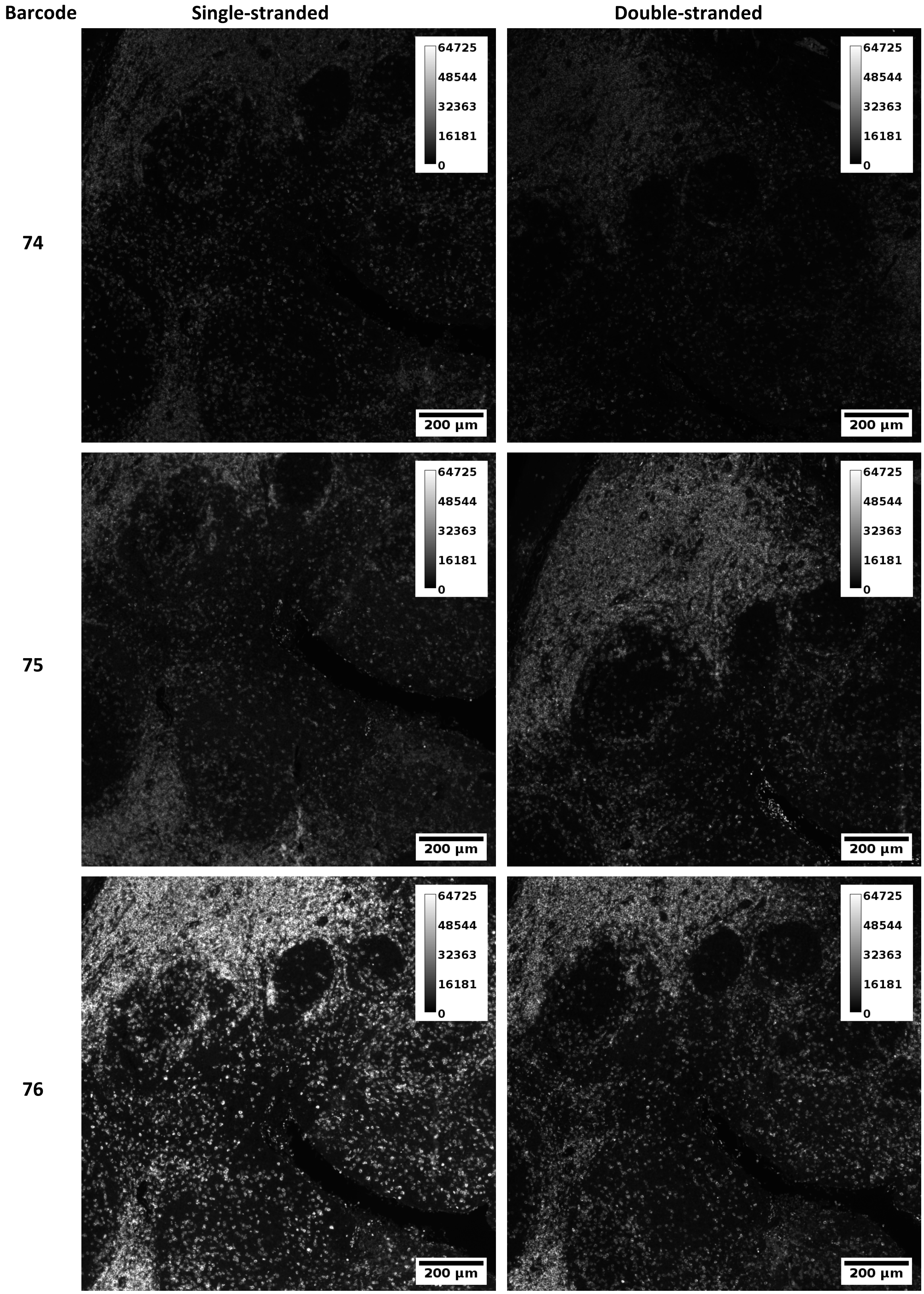

Supplementary Figure 14. Comparison of single-stranded versus double-stranded tyramide-barcode staining for barcodes labeled “74”, “75”, and “76”. See also Supplementary Figures 13 and 15. Intensity scale bars are in terms of camera counts.

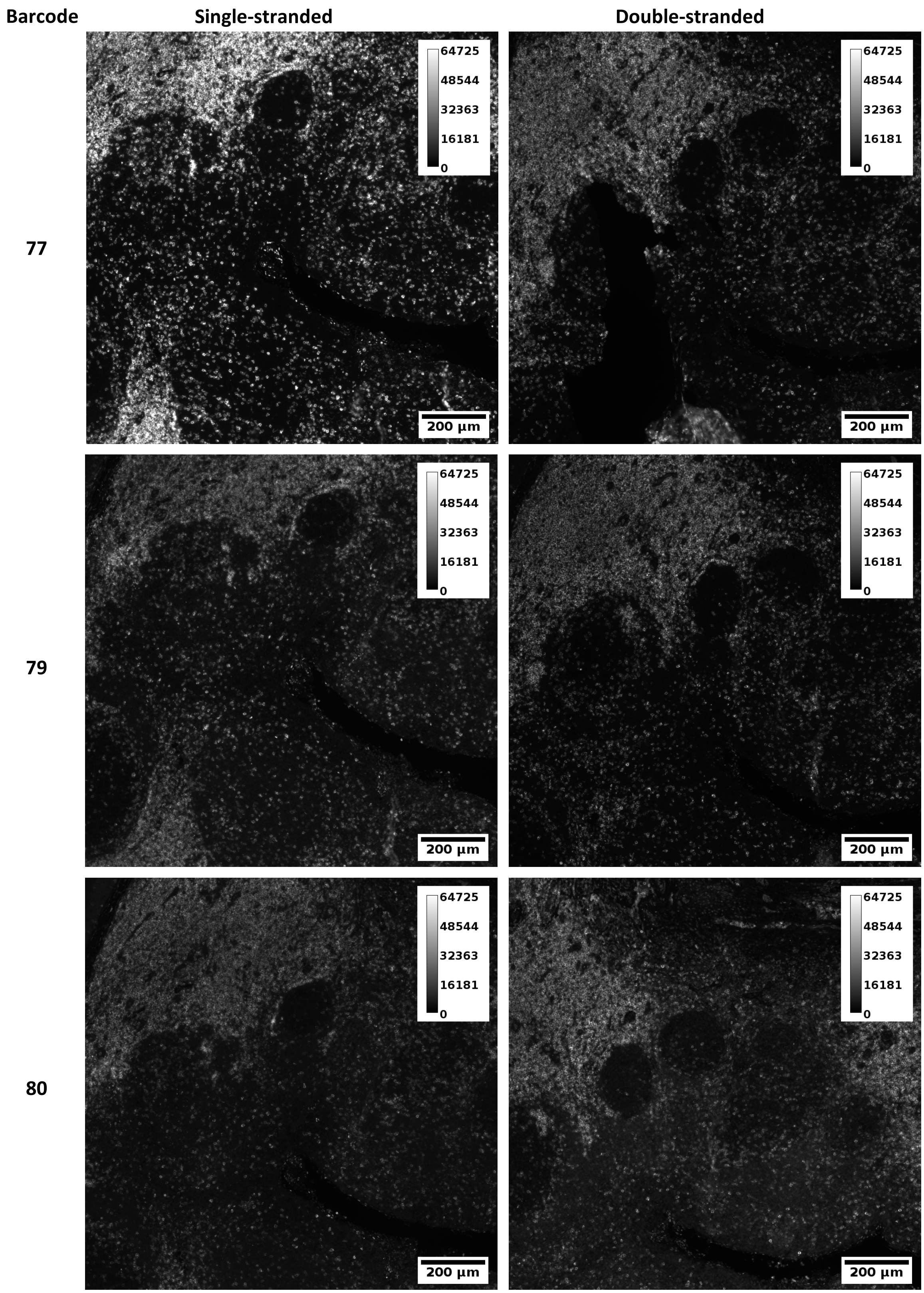

Supplementary Figure 15. Comparison of single-stranded versus double-stranded tyramide-barcode staining for barcodes labeled “77”, “79”, and “80”. See also Supplementary Figures 13 and 14. Intensity scale bars are in terms of camera counts.
