# Supplementary material for: Tyramide-conjugated DNA barcodes enable signal amplification for multiparametric CODEX imaging
Source: Commun Biol. 2022 Jun 27;5:627. doi: 10.1038/s42003-022-03558-8 (PMC9234042; doi:10.1038/s42003-022-03558-8)
Supplement: Supplementary file 3 — Reporting Summary [file 42003_2022_3558_MOESM3_ESM.pdf]

nature research

Corresponding author(s): Paul D. Simonson

Last updated by author(s): Apr 26, 2022

Reporting Summary

Nature Research wishes to improve the reproducibility of the work that we publish. This form provides structure for consistency and transparency in reporting. For further information on Nature Research policies, see our Editorial Policies and the Editorial Policy Checklist.

Statistics

For all statistical analyses, confirm that the following items are present in the figure legend, table legend, main text, or Methods section.

n/a	Confirmed	
☒	☐	The exact sample size (n) for each experimental group/condition, given as a discrete number and unit of measurement
☒	☐	A statement on whether measurements were taken from distinct samples or whether the same sample was measured repeatedly
☒	☐	The statistical test(s) used AND whether they are one- or two-sided<br>*Only common tests should be described solely by name; describe more complex techniques in the Methods section.*
☒	☐	A description of all covariates tested
☒	☐	A description of any assumptions or corrections, such as tests of normality and adjustment for multiple comparisons
☒	☐	A full description of the statistical parameters including central tendency (e.g. means) or other basic estimates (e.g. regression coefficient) AND variation (e.g. standard deviation) or associated estimates of uncertainty (e.g. confidence intervals)
☒	☐	For null hypothesis testing, the test statistic (e.g. F, t, r) with confidence intervals, effect sizes, degrees of freedom and P value noted<br>*Give P values as exact values whenever suitable.*
☒	☐	For Bayesian analysis, information on the choice of priors and Markov chain Monte Carlo settings
☒	☐	For hierarchical and complex designs, identification of the appropriate level for tests and full reporting of outcomes
☒	☐	Estimates of effect sizes (e.g. Cohen's d, Pearson's r), indicating how they were calculated

Our web collection on statistics for biologists contains articles on many of the points above.

Software and code

Policy information about availability of computer code

Data collection	CODEX Instrument Manager, CODEX Processor, ImageJ, CODEX MAV plugin for ImageJ, HALO, python, custom software found at https://github.com/SimonsonLab/VirtualHE_examples
Data analysis	Data were collected using the current (2021) commercial version of CODEX Instrument Manager (Akoya Biosciences). Post-image-collection processing was performed using the current (2021) CODEX Processor software (Akoya Biosciences). ImageJ (freely available) was used for additional image evaluation, with the CODEX MAV plugin (Akoya Bioscences). Images were also produced using commercially available HALO (2021 version). Custom software available at https://github.com/SimonsonLab/VirtualHE_examples was used to generate virtual H&E images (run using python).

For manuscripts utilizing custom algorithms or software that are central to the research but not yet described in published literature, software must be made available to editors and reviewers. We strongly encourage code deposition in a community repository (e.g. GitHub). See the Nature Research guidelines for submitting code & software for further information.

Data

Policy information about availability of data

All manuscripts must include a data availability statement. This statement should provide the following information, where applicable:

- Accession codes, unique identifiers, or web links for publicly available datasets
- A list of figures that have associated raw data
- A description of any restrictions on data availability

Imaging related data can be made available upon request.

Field-specific reporting

Please select the one below that is the best fit for your research. If you are not sure, read the appropriate sections before making your selection.

☒ Life sciences ☐ Behavioural & social sciences ☐ Ecological, evolutionary & environmental sciences

For a reference copy of the document with all sections, see nature.com/documents/nr-reporting-summary-flat.pdf

Life sciences study design

All studies must disclose on these points even when the disclosure is negative.

Sample size	The tyramide-barcode staining was performed in various samples, as already described in the paper.
Data exclusions	No exclusions.
Replication	The tyramide-barcode staining was replicated multiple times using multiple different antibodies, as described in the paper.
Randomization	Not applicable.
Blinding	Not applicable.

Reporting for specific materials, systems and methods

We require information from authors about some types of materials, experimental systems and methods used in many studies. Here, indicate whether each material, system or method listed is relevant to your study. If you are not sure if a list item applies to your research, read the appropriate section before selecting a response.

Materials & experimental systems

n/a	Involved in the study	
☐	☒	Antibodies
☒	☐	Eukaryotic cell lines
☒	☐	Palaeontology and archaeology
☒	☐	Animals and other organisms
☒	☐	Human research participants
☒	☐	Clinical data
☒	☐	Dual use research of concern

Methods

n/a	Involved in the study	
☒	☐	ChIP-seq
☒	☐	Flow cytometry
☒	☐	MRI-based neuroimaging

Antibodies

Antibodies used

Antibodies included those that were commercially pre-conjugated with DNA barcodes (Akoya Biosciences). We also used commercial antibodies for all other antibodies, which include:
Atibody Clone Vendor Catalog No.
CD3 SP7 Millipore Sigma SAB5500058
CD4 4B12 Leica NCL-L-CD4-368
CD10 OTI2A4 Origene CF810614
ICOS D1K2T Cell Signaling 89601
CD30 Ber-H2 Invitrogen MA5-13219
MUM1 MUM1p Dako(Agilent) M725901-2
CD163 10D6 NovusBio NB110-59935

Validation

Primary antibodies (not from Akoya Biosciences) were checked for appropriate staining using conventional DAB-based immunohistochemistry prior to running on the CODEX instrument. Appropriate staining was verified by board-certified pathologists.
