# Supplementary material for: Tyramide-conjugated DNA barcodes enable signal amplification for multiparametric CODEX imaging
Source: Commun Biol. 2022 Jun 27;5:627. doi: 10.1038/s42003-022-03558-8 (PMC9234042; doi:10.1038/s42003-022-03558-8)
Supplement: Supplementary file 4 — Description of Additional Supplementary Files [file 42003_2022_3558_MOESM4_ESM.pdf]

Description of Additional Supplementary Files

File Name: Supplementary Data 1
Description: Calculated marker intensities and population annotations listed in Figure 5 for each cell.
